# Epidemiological shift in age distribution of dengue in Thailand, 2005–2024

**DOI:** 10.1371/journal.pntd.0014697

**Published:** 2026-08-31

**Authors:** Kittipong Sornlorm, Roshan Kumar Mahato, Sarayu Muntaphan, Kanit Hnuploy, Rajitra Nawawonganun

**Affiliations:** 1 Khon Kaen University, Khon Kaen, Thailand; 2 Khon Kaen Provincial Public Health Office, Khon Kaen, Thailand; 3 Suratthani Rajabhat University, Surat Thani, Thailand; Air Force Medical University, CHINA

## Abstract

Dengue has historically been characterized as a childhood disease, but evidence of shifting age distribution is increasing. We analyzed 20 years (2005–2024) of national dengue surveillance data from Thailand comprising 1,639,442 cases to examine temporal trends in age-specific incidence, quantify age shift magnitude using the ratio of incidence rate ratios (RIRR), and decompose observed changes using Kitagawa decomposition. The proportion of cases among adults ≥25 years nearly doubled from 19.56% to 36.16%, while the children’s proportion declined from 54.49% to 41.63%. RIRR for adults 25–44 years was 1.78 (95% CI 1.76-1.80), and for older adults ≥45 years was 2.19 (95% CI 2.15-2.24). Kitagawa decomposition attributed +178.7% of change to epidemiological factors and -78.7% to demographic shifts, supporting a declining force of infection rather than population aging as the primary driver. These findings necessitate expanding dengue prevention beyond school-based programs and enhancing clinical vigilance for adult dengue.

## Author summary

Dengue has long been regarded as a childhood disease, and prevention in endemic countries such as Thailand has centered on schools. Using two decades of national surveillance data (2005–2024) covering more than 1.6 million dengue cases, we show that the age of people affected by dengue in Thailand has shifted substantially upward. The average age of cases rose from about 17 to 26 years, and the share of cases in adults aged 25 years and older nearly doubled, from roughly one-fifth to more than one-third. This upward shift was seen in almost every province and region and, using a demographic decomposition method, was driven mainly by changing infection risk at each age (a declining “force of infection”) rather than by aging of the population itself. These findings indicate that dengue prevention, clinical vigilance and vaccination planning in Thailand and similar settings must expand beyond children to protect the growing number of affected adults, while continuing to safeguard children, who still bear the largest share of cases.

## Introduction

Dengue is the most rapidly spreading arboviral disease globally, with an estimated 390 million infections occurring annually, of which approximately 96 million manifest clinically [1,2]. Over the past five decades, global dengue incidence has increased 30-fold, accompanied by geographic expansion into previously unaffected regions [3,4]. In 2024, a record 14.1 million cases were reported worldwide, representing a twofold increase compared with 2023 [5].

Southeast Asia accounts for approximately 70% of the global dengue burden [6]. Thailand, located at the epicenter of this endemic region, has experienced recurrent epidemics since the first major outbreak in 1958, with reported cases typically ranging from 50,000–150,000 annually and periodic surges exceeding 100,000 cases during epidemic years [7,8]. The country maintains a robust national surveillance system that has provided extensive longitudinal data on dengue epidemiology [9].

Historically, severe dengue has predominantly affected children, a pattern attributed to antibody-dependent enhancement during secondary heterotypic infections [10,11]. Consequently, dengue prevention programs have traditionally focused on school-based interventions and pediatric healthcare settings. However, increasing evidence from Thailand [12], Brazil [13], Singapore [14], Vietnam [15], and Puerto Rico [16] indicates an increasing disease burden among adult populations. Several mechanisms may explain this epidemiological transition. The force of infection hypothesis proposes that reduced transmission intensity, resulting from sustained vector control programs, delays primary infections to older ages [12,13]. Concurrent demographic transition with declining birth rates alters population age structure [17]. Additionally, changes in human mobility patterns and urbanization may modify exposure risks across age groups [18].

Understanding these trends is crucial because dengue in adults frequently presents with atypical manifestations [19,20], and adults with comorbidities face elevated risks of severe outcomes [21,22]. Furthermore, vaccine development and deployment strategies require accurate characterization of age-specific disease burden [5,23].

This study aimed to: (1) examine temporal trends in age-specific dengue incidence in Thailand using 20 years of national surveillance data (2005–2024); (2) estimate age-specific incidence rate ratios and their temporal changes using the ratio of IRRs (RIRR); and (3) decompose observed changes into demographic and epidemiological components using Kitagawa decomposition to elucidate the mechanisms driving shifts in dengue age distribution.

## Materials and methods

### Ethics statement

This study was reviewed by the Khon Kaen University Ethics Committee for Human Research and granted exemption from full ethical approval (Reference No. HE 692027).

### Study design and setting

National dengue surveillance data from Thailand were retrospectively analyzed for the 20-year period from January 1, 2005, to December 31, 2024 (Buddhist Era years 2548–2567). Thailand comprises approximately 66 million people across 77 provinces.

### Data sources and case definition

Dengue case data were obtained from the Bureau of Epidemiology, Department of Disease Control, Ministry of Public Health, Thailand. Cases were reported through the national disease surveillance system (Report 506) [24]. All healthcare facilities are required to report confirmed and probable cases. The surveillance data included all reported dengue cases comprising three clinical presentations: dengue fever (DF), dengue hemorrhagic fever (DHF), and dengue shock syndrome (DSS). These three clinical entities were analyzed collectively as total dengue cases, consistent with standard epidemiological practice and World Health Organization (WHO) recommendations for burden estimation [25]. Population denominators were obtained from the Official Statistics Registration Systems, Ministry of Interior. Mid-year population estimates stratified by age group and province were used to calculate age-specific incidence rates [26].

### Study variables

Age groups were categorized into four strata: children (0–14 years), youth (15–24 years; a descriptive label that does not denote legal minority), adults (25–44 years), and older adults (≥45 years). These bands match the fixed age groups in which Thailand’s national surveillance and population-registration data are released and align with those used in prior Thai and regional dengue age-distribution studies [8,12,16], facilitating direct comparison. Study periods were divided into four 5-year intervals (2005–2009, 2010–2014, 2015–2019, and 2020–2024) to align with policy-planning cycles and to reduce the large single-year fluctuations produced by dengue’s multi-year epidemic cycles. Because any categorization can obscure within-stratum patterns, we complemented these analyses with non-discretized analyses that treated calendar time as continuous and used finer age strata (see Statistical Analysis).

### Statistical analysis

**Fig 1:**
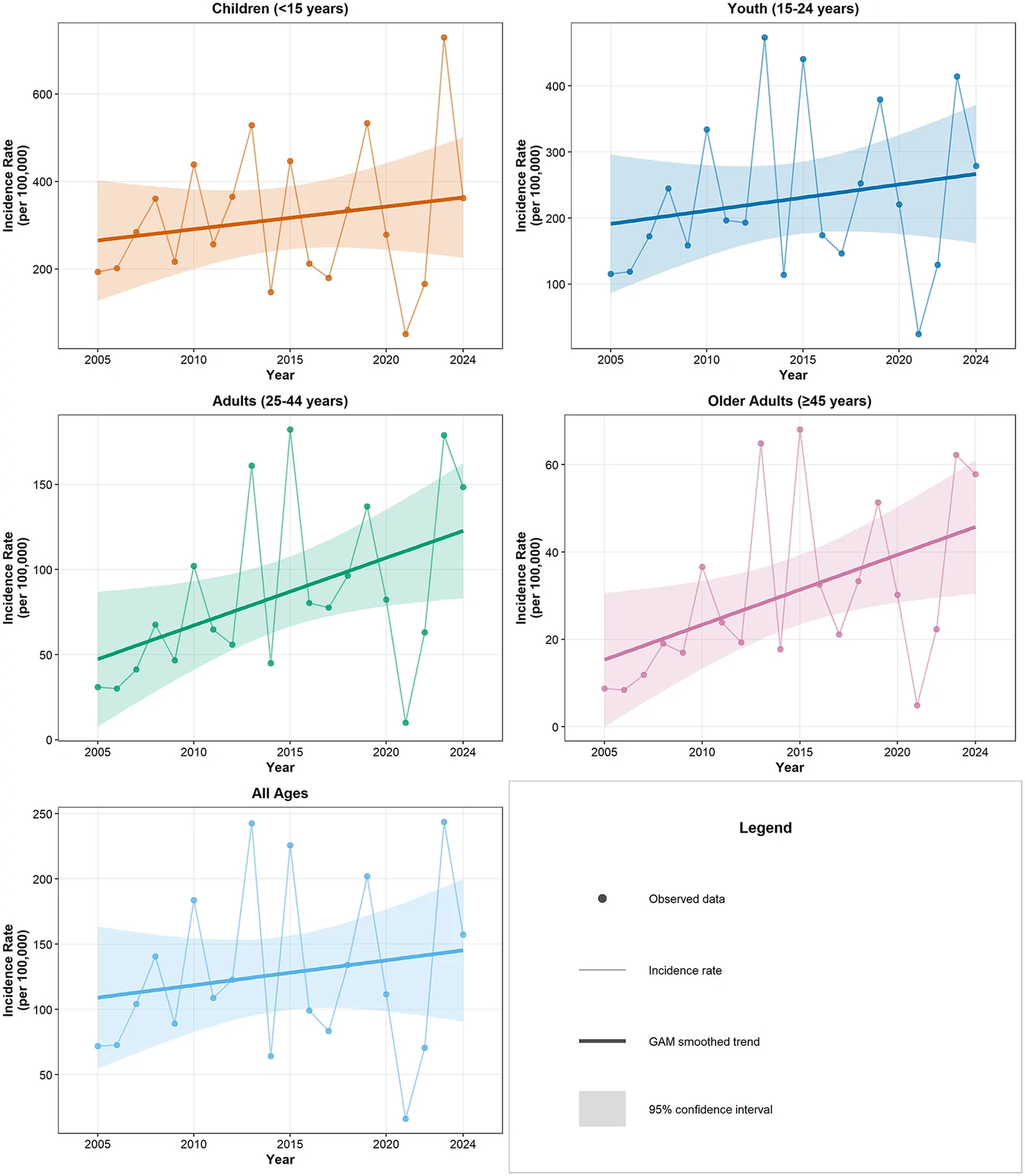
Age-specific dengue incidence trends with generalized additive model smoothing Thailand 2005-2024.

Age-specific incidence rates were calculated per 100,000 person-years. For visualization of temporal patterns, age-specific annual rates were smoothed with generalized additive models (GAMs; one model per age series) of the form rate ~ s(year, k = 8), fitted with a Gaussian family and identity link using penalized thin-plate regression splines, with the smoothing parameter selected by restricted maximum likelihood (REML) in the mgcv package [27]. Because annual rates are dominated by dengue’s epidemic cycles, the penalized fits were near-linear (effective degrees of freedom ≈ 2); per-series effective degrees of freedom, deviance explained and adjusted R^2^ are reported in the S1 Table legend, and the GAM curves (Fig 1) are presented as a descriptive summary, with formal inference based on the regression models described below. Trends in the age-group composition of cases were tested with Cochran-Armitage trend tests across the four ordered study periods (period scores 1–4), reported as the trend statistic Z with two-sided P [28]; these were complemented by tests of the annual (continuous-time) trend in the proportion of adult cases and in the mean age of cases.

Poisson regression models with a log-population offset were fitted to estimate incidence rate ratios (IRR). The ratio of IRRs (RIRR) quantified changes in relative risk between periods and was defined as:


$$\begin{array}{c} \text{RIRR }=\text{ IRR}({\text{Period}}_{\text{2}})\;/\text{ IRR}({\text{Period}}_{\text{1}}) \end{array}$$ (1)

where IRR represents the age-specific incidence rate ratio compared to children within each study period. Standard errors for ln(RIRR) were calculated using the delta method.

Kitagawa decomposition analysis [29,30] was employed to separate the change in crude incidence rate between periods into two distinct components: the rate effect (epidemiological component) and the composition effect (demographic component). The total change in crude rate (ΔR) between two time periods can be expressed as:


$$\begin{array}{c} \Delta \text{R}={\text{R}}_{\text{2}}-{\text{R}}_{\text{1}}=\text{Rate Effect}+\text{Composition Effect} \end{array}$$ (2)

The rate effect represents the change attributable to changes in age-specific incidence rates, holding population composition constant:


$$\begin{array}{c} \text{Rate Effect }=\;\Sigma_{\text{i}}\;[({\text{R}}_{\text{2}\text{i}}-{\text{R}}_{\text{1}\text{i}})\;\times \;{\overset{―}{\text{W}}}_{\text{i}}] \end{array}$$ (3)

The composition effect represents the change attributable to shifts in population age structure:


$$\begin{array}{c} \text{Composition Effect}=\Sigma_{\text{i}}\;[({\text{W}}_{\text{2}\text{i}}-{\text{W}}_{\text{1}\text{i}})\times {\overset{―}{\text{R}}}_{\text{i}}] \end{array}$$ (4)

where Rᵢ represents age-specific incidence rate for age group i, Wᵢ represents population weight (proportion) for age group i, subscripts 1 and 2 denote earlier and later periods respectively, ${\overset{―}{\text{W}}}_{\text{i}}$ = (W_1i_ + W_2i_)/2 is the average weight, and ${\overset{―}{\text{R}}}_{\text{i}}$ = (R_1i_ + R_2i_)/2 is the average rate.

Poisson regression models were fitted with a log-population offset. We estimated: (i) crude single-factor models for period and for age group; (ii) an adjusted main-effects model including both period and age group (reference categories 2005–2009 and children 0–14 years); (iii) a full model adding the period × age interaction, compared with the main-effects model by a likelihood-ratio test; and (iv) stratum-specific models within each period. Incidence rate ratios (IRRs) are reported with Wald 95% confidence intervals, and ratios of IRRs (RIRRs) with delta-method confidence intervals. Analyses were performed using StataNow 18.5 and R 4.x; figures were generated in R (ggplot2, mgcv) and Python (Matplotlib). Statistical significance was set at α = 0.05 and all *P* values are two-sided.

To assess whether the findings depended on the discretization of age and time, we performed three further sets of analyses. First, we computed the mean and median age of cases for each calendar year (using age-band midpoints) and estimated their linear trend over 2005–2024, and we modelled the annual proportion of cases aged ≥25 years the same way, by ordinary least-squares (OLS) regression on calendar year (Fig 3A, 3B). Because dengue incidence is strongly cyclical, we assessed first-order temporal autocorrelation in the OLS residuals using the Durbin-Watson statistic and confirmed the robustness of each trend by re-estimating it with heteroskedasticity- and autocorrelation-consistent (Newey-West) standard errors and with a Prais-Winsten AR(1) model. Second, we fitted a Poisson model to nine finer age strata (0–4, 5–9, 10–14, 15–24, 25–34, 35–44, 45–54, 55–64 and ≥65 years) with calendar year entered as a continuous covariate and an age × year interaction (offset = log population), yielding age-specific annual rate ratios and a likelihood-ratio test of the interaction. Third, to examine spatial generalizability, we computed the proportion of adult (≥25-year) cases by health region and by province for 2005–2009 versus 2020–2024 and tested the provincial trends.

**Fig 2:**
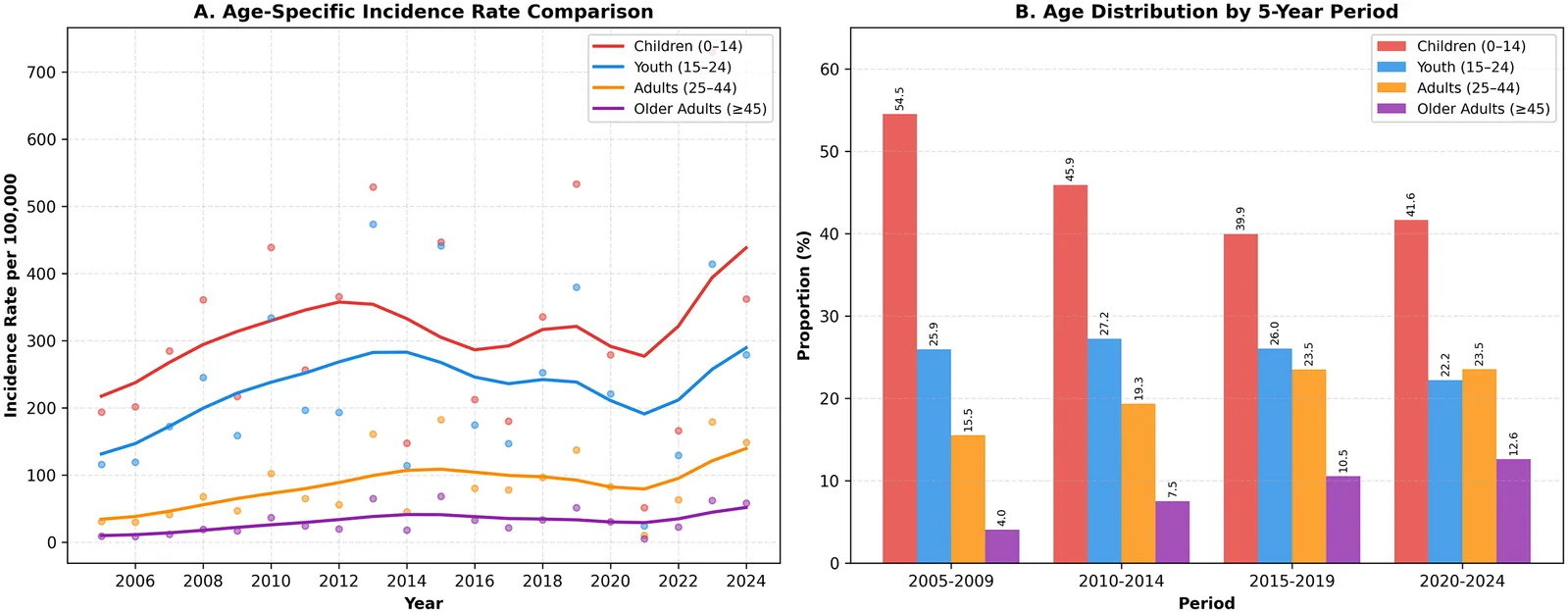
Changes in age distribution of dengue cases, Thailand 2005-2024.

## Results

### Descriptive characteristics

A total of 1,639,442 dengue cases were reported during the 20-year study period. Table 1 presents the distribution by period and age group. Children aged <15 years accounted for 733,050 cases (44.71%), with incidence rates ranging from 251.16 per 100,000 (2005–2009) to 348.05 per 100,000 (2010–2014). Youth 15–24 years accounted for 416,983 cases (25.44%), adults 25–44 years for 341,940 cases (20.86%), and older adults ≥45 years for 147,469 cases (9.00%). Cochran-Armitage trend tests revealed statistically significant linear trends for all age groups (Table 1, footnote). The proportion of children showed a highly significant decreasing trend (Z = -115.77, *P* < 0.001), as did youth (Z = -41.30, *P* < 0.001). Both adult groups demonstrated significant increasing trends: adults 25–44 years (Z = +91.76, *P* < 0.001) and older adults ≥45 years (Z = +133.74, *P* < 0.001).

**Table 1:** Distribution and trend analysis of dengue cases by period and age group, Thailand, 2005-2024.

Period	Children 0-14	Youth 15-24	Adults 25-44	Older Adults ≥45	Total
**2005-2009**					
Cases (n)	165,860	78,969	47,244	12,291	**304,364**
% within Period	54.49	25.95	15.52	4.04	100.00
Person-years	66,037,141	48,748,171	109,060,167	94,310,086	318,155,565
Rate	251.16	161.99	43.32	13.03	95.67
**2010-2014**					
Cases (n)	211,033	125,253	88,968	34,654	**459,908**
% within Period	45.89	27.23	19.34	7.54	100.00
Person-years	60,633,279	47,720,089	103,784,716	106,569,812	318,707,896
Rate	348.05	262.47	85.72	32.52	144.30
**2015-2019**					
Cases (n)	192,042	125,188	112,990	50,695	**480,915**
% within Period	39.93	26.03	23.50	10.54	100.00
Person-years	56,396,175	44,870,575	98,380,179	123,272,608	322,919,537
Rate	340.52	279.00	114.85	41.12	148.93
**2020-2024**					
Cases (n)	164,115	87,573	92,738	49,829	**394,255**
% within Period	41.63	22.21	23.52	12.64	100.00
Person-years	52,061,085	41,223,910	96,193,015	138,402,588	327,880,598
Rate	315.24	212.43	96.41	36.00	120.24
**Total (2005–2024)**					
Cases (n)	**733,050**	**416,983**	**341,940**	**147,469**	**1,639,442**
% of Total	44.71	25.43	20.86	9.00	100.00
Person-years	235,127,680	182,562,745	407,418,077	462,555,094	1,287,663,596
Overall Rate	311.77	228.41	83.93	31.88	127.32
**Trend Analysis**					
Absolute change	-12.86	-3.74	+8.00	+8.60	–
Z-statistic	-115.77	-41.30	+91.76	+133.74	–
*P*-value	<0.001	<0.001	<0.001	<0.001	–
Trend direction	Decreasing	Decreasing	Increasing	Increasing	–

Note. Rate, cases per 100,000 person-years. Z and *P* are from Cochran-Armitage tests for trend in the age-group proportion of cases across the four ordered periods (period scores 1–4). pp, percentage points.

**Table 2:** Incidence Rate Ratios from Poisson regression analysis, Thailand, 2005-2024.

Variable	Cases (n)	Person-years	Rate*	Crude IRR	Adjusted IRR†	95% CI	*P*-value
**Period**							
2005-2009	304,364	318,155,565	95.67	Ref.	Ref.	–	–
2010-2014	459,908	318,707,896	144.30	1.51	1.58	1.58-1.59	<0.001
2015-2019	480,915	322,919,537	148.93	1.56	1.73	1.73-1.74	<0.001
2020-2024	394,255	327,880,598	120.24	1.26	1.49	1.49-1.50	<0.001
**Age Group**							
Children 0–14	733,050	235,127,680	311.77	Ref.	Ref.	–	–
Youth 15–24	416,983	182,562,745	228.41	0.73	0.73	0.73-0.73	<0.001
Adults 25–44	341,940	407,418,077	83.93	0.27	0.27	0.27-0.27	<0.001
Older Adults ≥45	147,469	462,555,094	31.88	0.10	0.10	0.10-0.10	<0.001

Note. *Rate per 100,000 person-years. †Adjusted incidence rate ratio from a Poisson model including both period and age group (reference categories: period 2005–2009 and children 0–14 years). IRR, incidence rate ratio; CI, confidence interval. All *P*-values are two-sided.

### Changes in age distribution

The proportion of cases in each age group changed substantially over the study period (Table 1). Fig 1 presents age-specific incidence trends with GAM smoothing across the four analysis categories, showing trends for children (<15 years), youth (15–24 years), adults (25–44 years), older adults (≥45 years), and all ages combined. All age groups exhibited cyclical patterns corresponding to epidemic years, but with differential growth trajectories (S1 Table).

Fig 2 illustrates these proportional changes across periods. The proportion among children (<15 years) decreased consistently from 54.49% in 2005–2009 to 41.63% in 2020–2024, representing an absolute reduction of 12.86 percentage points. The proportion among youth (15–24 years) showed a slight decline from 25.95% to 22.21%. In contrast, the proportion among adults (25–44 years) increased from 15.52% to 23.52% (+8.00 percentage points), and older adults (≥45 years) showed the largest relative increase from 4.04% to 12.64% (+8.60 percentage points).

### Evidence of age shift

Table 2 presents crude IRRs. Compared to 2005–2009, all subsequent periods showed significantly higher incidence: 2010–2014 (IRR = 1.51, 95% CI: 1.50-1.52), 2015–2019 (IRR = 1.56, 95% CI: 1.55-1.56), and 2020–2024 (IRR = 1.26, 95% CI: 1.25-1.26). Compared to children, all other age groups had significantly lower rates: youth (IRR = 0.73), adults (IRR = 0.27), and older adults (IRR = 0.10). Adjusted IRRs were estimated from Poisson regression controlling for both period and age group. The period 2015–2019 showed the highest adjusted incidence (aIRR = 1.73, 95% CI: 1.73-1.74). Age effects remained consistent after adjustment.

The interaction between period and age group was highly statistically significant in the likelihood-ratio test (χ^2^ = 19,539.29, df = 9, P < 0.001), providing strong evidence that the relationship between age and dengue risk changed over time. Table 3 presents age-specific IRRs within each period. The gap between children and adults narrowed markedly from 2005–2009 to 2015–2019 (adults 25–44 years, IRR 0.17 to 0.34; older adults ≥45 years, 0.05 to 0.12), with a partial attenuation in 2020–2024 (adults 0.31; older adults 0.11); the 2020–2024 ratios nonetheless remained well above their 2005–2009 baseline, so the overall narrowing was sustained across the study period.

**Table 3:** Age-specific Incidence Rate Ratios by period, Thailand, 2005-2024.

Period	Age Group	Cases (n)	Person-years	Rate*	IRR	95% CI	*P*-value
**2005-2009**	Children 0–14	165,860	66,037,141	251.16	Ref.	–	–
	Youth 15–24	78,969	48,748,171	161.99	0.65	0.64-0.65	<0.001
	Adults 25–44	47,244	109,060,167	43.32	0.17	0.17-0.17	<0.001
	Older Adults ≥45	12,291	94,310,086	13.03	0.05	0.05-0.05	<0.001
**2010-2014**	Children 0–14	211,033	60,633,279	348.05	Ref.	–	–
	Youth 15–24	125,253	47,720,089	262.47	0.75	0.75-0.76	<0.001
	Adults 25–44	88,968	103,784,716	85.72	0.25	0.24-0.25	<0.001
	Older Adults ≥45	34,654	106,569,812	32.52	0.09	0.09-0.09	<0.001
**2015-2019**	Children 0–14	192,042	56,396,175	340.52	Ref.	–	–
	Youth 15–24	125,188	44,870,575	279.00	0.82	0.81-0.83	<0.001
	Adults 25–44	112,990	98,380,179	114.85	0.34	0.33-0.34	<0.001
	Older Adults ≥45	50,695	123,272,608	41.12	0.12	0.12-0.12	<0.001
**2020-2024**	Children 0–14	164,115	52,061,085	315.24	Ref.	–	–
	Youth 15–24	87,573	41,223,910	212.43	0.67	0.67-0.68	<0.001
	Adults 25–44	92,738	96,193,015	96.41	0.31	0.30-0.31	<0.001
	Older Adults ≥45	49,829	138,402,588	36.00	0.11	0.11-0.12	<0.001

Note. *Rate per 100,000 person-years. Reference category within each period: children 0–14 years. IRR, incidence rate ratio; CI, confidence interval. All *P*-values are two-sided.

Table 4 presents the RIRR comparing 2020–2024 with 2005–2009. The RIRR for youth was 1.04 (95% CI: 1.03-1.06, *P* < 0.001), indicating a modest 4% increase in relative risk. For adults 25–44 years, RIRR was 1.78 (95% CI: 1.76-1.80, *P* < 0.001), representing a 78% increase in relative risk compared to children. The largest increase was observed among older adults with RIRR of 2.19 (95% CI: 2.15-2.24, *P* < 0.001), indicating a 119% increase in relative risk.

**Table 4:** Ratio of Incidence Rate Ratios comparing 2020-2024 to 2005-2009, Thailand.

Age Group	IRR (2005-2009)	IRR (2020-2024)	RIRR	95% CI	*P*-value
Youth 15–24	0.65	0.67	1.04	1.03-1.06	<0.001
Adults 25–44	0.17	0.31	1.78	1.76-1.80	<0.001
Older Adults ≥45	0.05	0.11	2.19	2.15-2.24	<0.001

Note. RIRR, ratio of incidence rate ratios (IRR in 2020–2024 divided by IRR in 2005–2009, each relative to children 0–14 years); CI, confidence interval (delta method). All *P*-values are two-sided.

### Decomposition analysis

Kitagawa decomposition provided mechanistic insights by separating observed rate changes into distinct components (Table 5). The epidemiological component (rate effect) contributed +43.91 per 100,000 (+178.7% of total change), while the demographic component (composition effect) contributed -19.33 per 100,000 (-78.7%). This indicates that changes in age-specific disease risk, rather than population aging, were the primary drivers of the observed overall increase. Adults 25–44 years contributed the largest share to the rate effect (+16.89 per 100,000), followed by children (+11.74), older adults (+8.25), and youth (+7.03). The positive rate effect exceeding 100% indicates that true changes in age-specific disease risk were the primary driver of the epidemiological shift.

**Table 5:** Kitagawa decomposition of changes in crude dengue incidence rate between 2005-2009 and 2020-2024.

Age Group	Rate (2005-09)	Rate (2020-24)	Change (% change)	Rate Effect	Composition Effect
Children 0–14	251.16	315.24	+64.07 (+25.5%)	+11.74	-13.81
Youth 15–24	161.99	212.43	+50.44 (+31.1%)	+7.03	-5.15
Adults 25–44	43.32	96.41	+53.09 (+122.6%)	+16.89	-3.45
Older Adults ≥45	13.03	36.00	+22.97 (+176.3%)	+8.25	+3.08
TOTAL	95.67*	120.24*	+24.58	+43.91	-19.33
% of Total Change	–	–	100%	+178.7%	-78.7%

Note. *Crude all-age incidence rate per 100,000 person-years. The rate effect (epidemiological component) and composition effect (demographic component) sum to the total change; percentages are expressed relative to the total change in the crude rate.

### Robustness to age and time discretization

**Fig 3:**
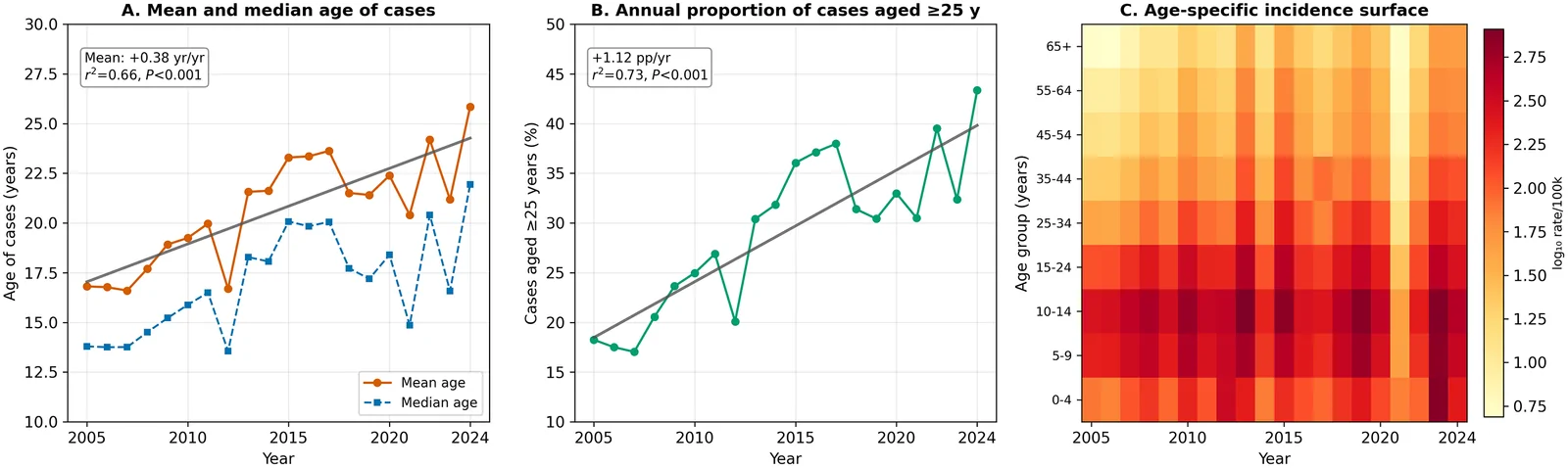
Non-discretized evidence of the dengue age shift, Thailand 2005-2024. **(A)** Mean and median age of reported cases per year with linear trends. **(B)** Annual proportion of cases aged ≥25 years with linear trend. **(C)** Age-specific incidence-rate surface (log₁₀ rate per 100,000) across nine age strata and calendar year.

To ensure these findings did not depend on the chosen categories, we repeated the analysis without discretizing age or time (Fig 3). The mean age of reported cases rose steadily from 16.8 years in 2005 to 25.9 years in 2024 (linear trend +0.38 years per calendar year; 95% CI 0.26-0.51; r^2^ = 0.66; P < 0.001) and the median age from 13.8 to 21.9 years (+0.30 years/year; P < 0.001), with results insensitive to the assumed midpoint of the open-ended ≥65-year band (Fig 3A). The annual proportion of cases aged ≥25 years increased from 18.2% to 43.4% (+1.12 percentage points/year; r^2^ = 0.73; P < 0.001), a monotonic trend accounting for nearly three-quarters of the year-to-year variance and indicating a structural shift rather than short-term fluctuation (Fig 3B). Because dengue incidence is strongly cyclical, we confirmed that this trend was not an artif**a**ct of temporal autocorrelation: residual autocorrelation was low (Durbin-Watson = 1.54 for the ≥ 25-year proportion and 1.86 for mean age; lag-1 residual autocorrelation ≤ 0.22) and the estimated slope was essentially unchanged under autocorrelation-consistent inference (Newey-West and Prais-Winsten AR(1): + 1.12 and +1.14 percentage points/year for the ≥ 25-year proportion, respectively; both P < 0.001). The age-year incidence surface displays this transition directly: incidence in the oldest strata warmed progressively across the study period, whereas the school-age strata remained comparatively stable (Fig 3C). Consistent with this visualization, a Poisson model using nine finer age strata with calendar year as a continuous covariate showed incidence increasing most steeply in the oldest strata (≥65 years, + 7.8%/year; 55–64, + 5.3%; 35–44, + 5.4%; 25–34, + 4.7%) and least in the school-age strata (5–9, + 1.1%; 10–14, + 0.8%); the age × (continuous) year interaction was highly significant (χ^2^ = 18,873, df = 8, P < 0.001), and the ratio of older-adult (≥45 years) to child (<15 years) incidence rose from 0.045 in 2005 to 0.160 in 2024.

### Geographical consistency of the age shift

**Fig 4:**
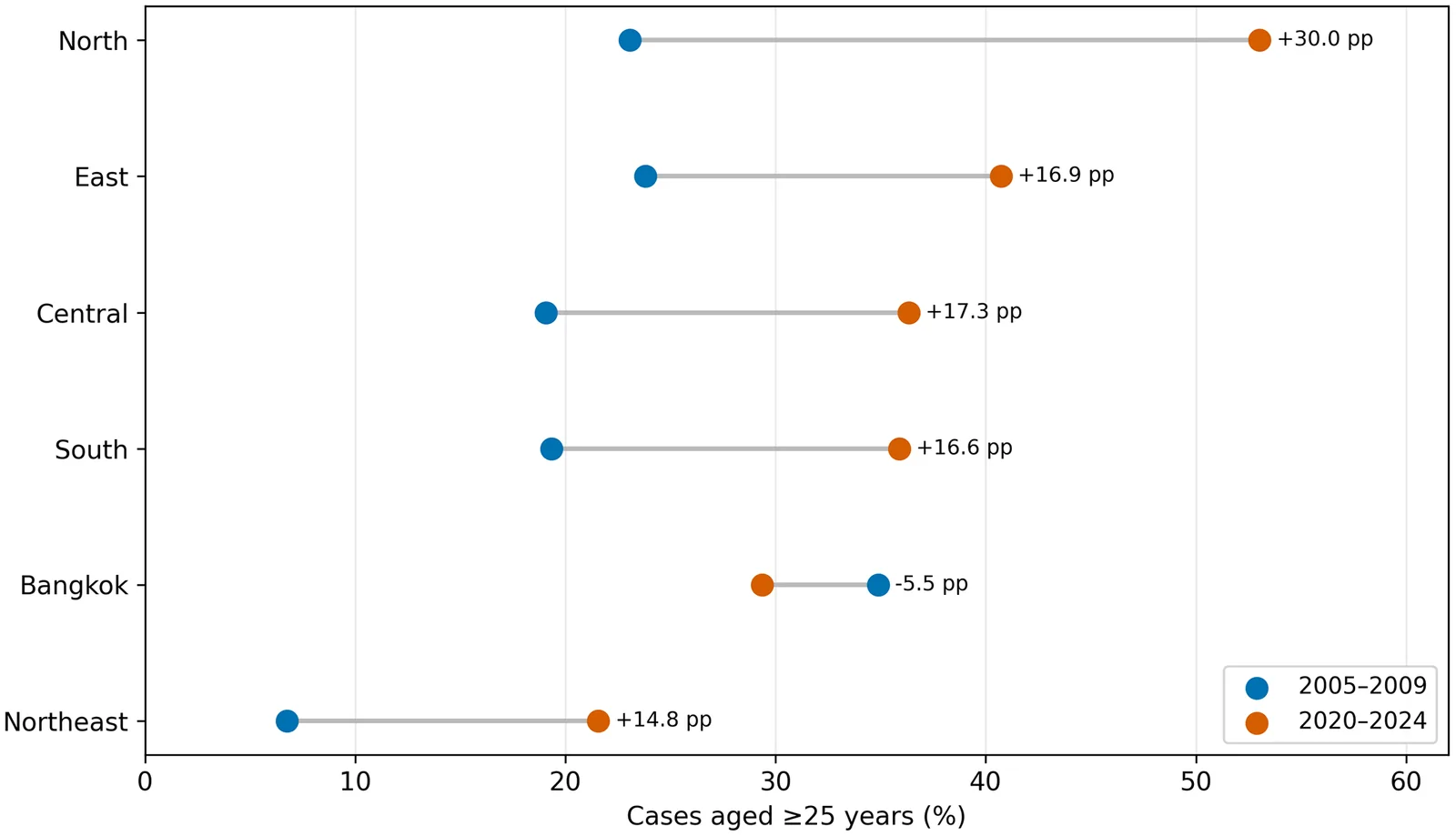
Proportion of dengue cases aged ≥25 years by region, 2005-2009 versus 2020-2024, Thailand. Each region’s change (percentage points) is annotated; the adult proportion increased in every region except Bangkok.

The upward age shift was geographically pervasive rather than confined to particular areas (Fig 4). The proportion of adult (≥25-year) cases increased in five of six regions with data in both periods (all provincial trends *P* < 0.001; S2 Table): North +30.0, Central +17.3, East +16.9, South +16.6, and Northeast +14.8 percentage points. The single exception was Bangkok, the most urbanized setting, where the adult proportion was already highest at baseline (34.9%) and changed little (-5.5 percentage points). Large increases even in predominantly rural regions such as the Northeast are more consistent with a general decline in the force of infection than with expansion of transmission into a limited number of new locales.

## Discussion

This comprehensive analysis of 20 years of national dengue surveillance data from Thailand provides robust evidence of a significant and sustained upward shift in the age distribution of dengue cases. The relative risk among adults 25–44 years increased by 78% and among older adults ≥45 years by 119% when comparing 2020-2024 with 2005-2009. These findings extend earlier observations by Cummings et al. [12] using an additional 15 years of data and demonstrate a continued epidemiological transition.

The proportion of cases among adults ≥25 years nearly doubled from 19.56% to 36.16% over the study period. GAM analysis revealed distinct temporal patterns across age groups, with children showing cyclical fluctuations around a relatively stable mean, whereas adult groups demonstrated clearer upward trajectories. This differential pattern was confirmed statistically through the significant period and age interaction. Similar age shifts have been reported from Puerto Rico, where the median age of cases increased from 19 years (2010–2019) to 26 years (2023–2024) [16].

The Kitagawa decomposition analysis provides novel mechanistic insight. The finding that epidemiological factors accounted for +178.7% of total change, while demographic factors contributed -78.7%, strongly supports the force of infection hypothesis. This indicates that reduced transmission intensity has delayed primary infections to older ages, rather than population aging being the primary driver of the observed shift. Notably, all age groups showed increased incidence rates over time, but with differential magnitudes that altered the proportional distribution.

The consistency of the shift across analytical choices strengthens these conclusions. Continuous-time analyses (the rising mean age of cases and the monotonic increase in the adult proportion) show that the pattern is a structural, decades-long transition rather than an artifact of age or period categorization or of short-term epidemic fluctuation. Its geographical pervasiveness, affecting almost every province and all regions except the already-mature Bangkok setting, argues against the alternative explanation that rising adult incidence merely reflects the spread of dengue into new, less urban areas, and instead supports a widespread decline in the force of infection. A dedicated spatial and spatiotemporal analysis of these dynamics is the subject of ongoing companion work.

The partial attenuation of the child-adult gap in 2020–2024 warrants interpretation in light of the concurrent COVID-19 pandemic. During 2020–2021, movement restrictions, reduced workplace and community mobility, and disrupted care-seeking coincided with a fall in recorded all-age incidence to a nadir of 16.4 per 100,000 in 2021-the lowest of the study period-followed by resurgence to 243.7 per 100,000 in 2023. Because the exposure of working-age adults is comparatively more dependent on movement outside the home, these disruptions plausibly dampened the adult signal transiently, consistent with the modest dip in adult IRRs in 2020–2024 (0.31, versus 0.34 in 2015–2019). Crucially, the upward age shift was already established before the pandemic (through 2015–2019), and the annual proportion of cases aged ≥25 years reached its highest value (43.4%) in 2024; COVID-19 therefore appears to have temporarily perturbed, rather than driven or reversed, the longer-term transition.

These findings align with regional patterns observed in Singapore [14], Vietnam [15], and Brazil [13] and Puerto Rico [16]. The consistency across diverse settings suggests that the age shift represents a generalizable consequence of improved vector control and changing transmission dynamics in transitioning endemic settings, and global burden estimates likewise document rising dengue incidence among adults aged 20–49 years [31].

These trends carry direct clinical and public-health implications for Thailand. Because adult dengue may present as undifferentiated febrile illness overlapping with conditions such as leptospirosis, scrub typhus and influenza, and adults with comorbidities face elevated risks of severe outcomes [19–22], clinicians should maintain heightened suspicion for dengue in adults and hospitals should consider revising emergency-department triage during epidemic periods. At the program level, the national control effort’s traditional emphasis on school-based Aedes larval source reduction, while still valuable, is increasingly insufficient to reach the growing adult caseload, so community- and workplace-based vector control is needed. The shift also bears on vaccination strategy: the tetravalent vaccine TAK-003 (Qdenga), which has shown 61% efficacy against virologically confirmed dengue and 84% against hospitalization and is recommended regardless of prior dengue exposure status [5,23], is well suited to the expanding adult-at-risk population, and age-stratified cost-effectiveness analyses incorporating the shifting age distribution may help optimize program design.

Importantly, this evolving epidemiology represents an expanding, dual burden rather than a transfer of risk away from children. Children (<15 years) still constituted the single largest group (44.7% of all cases over the study period) and continue to require intensive prevention. School-based vector control therefore remains essential and should be complemented, not replaced, by community-, workplace- and adult-focused measures.

Several limitations should be considered when interpreting these findings. The analysis is ecological and based on aggregate surveillance data, precluding adjustment for individual-level risk factors, and the surveillance system may underestimate true incidence owing to subclinical infections, changes in diagnostic practice over time, and variation in healthcare-seeking behavior [8,32]. The national Report 506 system reports the three clinical entities-dengue fever, dengue hemorrhagic fever and dengue shock syndrome-in combination, and clinical-entity- and laboratory-confirmation-specific age breakdowns were not available for most of the study period; we therefore analysed all reported dengue collectively. This precludes determining whether the age shift differs by clinical severity, and any divergence in the age trajectories of severe (DHF/DSS) versus non-severe (DF) disease would be masked in the combined series; severity-specific surveillance would be required to resolve this. Further limitations include the use of categorical age and period definitions, although our continuous and finer-stratum analyses yielded concordant results; the unequal widths of the age bands; and the absence of formal spatial modelling, which we are addressing in dedicated companion work.

## Conclusions

The age distribution of dengue in Thailand has shifted significantly toward older age groups over two decades, with the relative risk among adults more than doubling compared with children, even as children continued to bear the largest single share of cases. Kitagawa decomposition demonstrates that this transition is driven primarily by epidemiological factors, specifically a reduced force of infection delaying primary infections, rather than by demographic population aging, and the shift was reproduced by non-discretized analyses and was geographically pervasive. These findings necessitate expansion of dengue prevention beyond traditional school-based programs, enhanced clinical vigilance for adult dengue presentations, and age-informed vaccination planning, while sustaining the school-based measures that remain essential for children.

## Supporting information

S1 Table Annual age-specific dengue incidence rates and generalized additive model (GAM) estimates, Thailand, 2005–2024. The legend reports per-series effective degrees of freedom, deviance explained and adjusted R^2^.
(DOCX)

S2 Table Adult (≥25-year) dengue case proportion by region and province, 2005–2009 vs 2020–2024. (DOCX)

S1 Data Aggregated dengue case counts and mid-year population denominators by year, 5-year period, age group, finer age band, health region and province, Thailand, 2005–2024 - the complete dataset analysed in this study. (XLSX)
