# Supplementary material for: Epidemiological shift in age distribution of dengue in Thailand, 2005–2024
Source: PLoS Negl Trop Dis. 2026 Aug 31;20(8):e0014697. doi: 10.1371/journal.pntd.0014697 (PMC13552936; doi:10.1371/journal.pntd.0014697)
Supplement: Supplementary file 1 [file pntd.0014697.s001.docx]

**S1 Table. Annual age-specific dengue incidence rates and generalized additive model (GAM) estimates, Thailand, 2005–2024.**

Legend. Rate, observed incidence per 100,000 person-years; GAM Fit and 95% CI, fitted values from rate ~ s(year, *k* = 8), Gaussian family, REML (mgcv). Per-series GAM diagnostics (effective degrees of freedom [edf], deviance explained, adjusted *R*²): children <15 y (edf 2, 3.6%, −0.018); youth 15–24 y (2, 3.7%, −0.017); adults 25–44 y (2, 21.0%, 0.166); older adults ≥45 y (2, 22.4%, 0.181); all ages (2, 3.1%, −0.023). The near-linear fits (edf ≈ 2) reflect the dominance of epidemic cycles; Figure 1 is descriptive and formal inference rests on the Poisson/RIRR/decomposition models.

This table presents annual dengue incidence rates per 100,000 person-years stratified by four age groups (0–14, 15–24, 25–44, and ≥45 years) in Thailand from 2005 to 2024.

Observed crude incidence rates are shown alongside fitted values derived from generalized additive models (GAMs) to illustrate non-linear temporal trends.

Smoothed estimates were generated using penalized regression splines with restricted maximum likelihood (REML) estimation.

These data correspond to Figure 1 and support interpretation of age-specific trend differentials underlying the epidemiological age shift.

| **Age Group** | **Year** | **Cases** | **Population** | **Rate** | **SE** | **GAM Fit** | **GAM SE** | **CI Lower** | **CI Upper** |
| --- | --- | --- | --- | --- | --- | --- | --- | --- | --- |
| <15 | 2005 | 26076 | 13465243 | 193.65 | 1.2 | 265.37 | 70.32 | 127.55 | 403.19 |
| <15 | 2006 | 26951 | 13369958 | 201.58 | 1.23 | 270.55 | 64.99 | 143.17 | 397.92 |
| <15 | 2007 | 37648 | 13230177 | 284.56 | 1.47 | 275.72 | 59.86 | 158.4 | 393.04 |
| <15 | 2008 | 47207 | 13080018 | 360.91 | 1.66 | 280.89 | 54.98 | 173.13 | 388.64 |
| <15 | 2009 | 27978 | 12891745 | 217.02 | 1.3 | 286.06 | 50.42 | 187.24 | 384.88 |
| <15 | 2010 | 55624 | 12672934 | 438.92 | 1.86 | 291.23 | 46.28 | 200.53 | 381.94 |
| <15 | 2011 | 32056 | 12496939 | 256.51 | 1.43 | 296.41 | 42.68 | 212.75 | 380.06 |
| <15 | 2012 | 43677 | 11959619 | 365.2 | 1.75 | 301.58 | 39.77 | 223.64 | 379.52 |
| <15 | 2013 | 62428 | 11804488 | 528.85 | 2.12 | 306.75 | 37.7 | 232.86 | 380.64 |
| <15 | 2014 | 17248 | 11699299 | 147.43 | 1.12 | 311.92 | 36.62 | 240.14 | 383.7 |
| <15 | 2015 | 51645 | 11557397 | 446.86 | 1.97 | 317.09 | 36.62 | 245.31 | 388.87 |
| <15 | 2016 | 24272 | 11433331 | 212.29 | 1.36 | 322.27 | 37.7 | 248.37 | 396.16 |
| <15 | 2017 | 20332 | 11304871 | 179.85 | 1.26 | 327.44 | 39.77 | 249.49 | 405.38 |
| <15 | 2018 | 37407 | 11153397 | 335.39 | 1.73 | 332.61 | 42.68 | 248.96 | 416.26 |
| <15 | 2019 | 58386 | 10947179 | 533.34 | 2.21 | 337.78 | 46.28 | 247.07 | 428.49 |
| <15 | 2020 | 29894 | 10721801 | 278.82 | 1.61 | 342.95 | 50.42 | 244.13 | 441.77 |
| <15 | 2021 | 5357 | 10453779 | 51.24 | 0.7 | 348.12 | 54.98 | 240.37 | 455.88 |
| <15 | 2022 | 17584 | 10606364 | 165.79 | 1.25 | 353.3 | 59.86 | 235.98 | 470.62 |
| <15 | 2023 | 75105 | 10292442 | 729.71 | 2.66 | 358.47 | 64.99 | 231.09 | 485.85 |
| <15 | 2024 | 36175 | 9986699 | 362.23 | 1.9 | 363.64 | 70.32 | 225.82 | 501.46 |
| 15-24 | 2005 | 11389 | 9855988 | 115.55 | 1.08 | 191.31 | 53.36 | 86.72 | 295.91 |
| 15-24 | 2006 | 11628 | 9764685 | 119.08 | 1.1 | 195.3 | 49.31 | 98.65 | 291.96 |
| 15-24 | 2007 | 16727 | 9711468 | 172.24 | 1.33 | 199.29 | 45.42 | 110.27 | 288.3 |
| 15-24 | 2008 | 23966 | 9784592 | 244.94 | 1.58 | 203.28 | 41.71 | 121.53 | 285.03 |
| 15-24 | 2009 | 15259 | 9631438 | 158.43 | 1.28 | 207.26 | 38.25 | 132.29 | 282.23 |
| 15-24 | 2010 | 32083 | 9607579 | 333.93 | 1.86 | 211.25 | 35.11 | 142.43 | 280.07 |
| 15-24 | 2011 | 18971 | 9652998 | 196.53 | 1.43 | 215.23 | 32.38 | 151.75 | 278.7 |
| 15-24 | 2012 | 18405 | 9521800 | 193.29 | 1.42 | 219.21 | 30.18 | 160.06 | 278.36 |
| 15-24 | 2013 | 45041 | 9510850 | 473.57 | 2.23 | 223.19 | 28.61 | 167.1 | 279.27 |
| 15-24 | 2014 | 10753 | 9426862 | 114.07 | 1.1 | 227.16 | 27.8 | 172.67 | 281.65 |
| 15-24 | 2015 | 41039 | 9309510 | 440.83 | 2.18 | 231.14 | 27.8 | 176.65 | 285.62 |
| 15-24 | 2016 | 15920 | 9140544 | 174.17 | 1.38 | 235.11 | 28.61 | 179.02 | 291.19 |
| 15-24 | 2017 | 13140 | 8959966 | 146.65 | 1.28 | 239.08 | 30.18 | 179.93 | 298.23 |
| 15-24 | 2018 | 22228 | 8798082 | 252.65 | 1.69 | 243.04 | 32.38 | 179.57 | 306.52 |
| 15-24 | 2019 | 32861 | 8662473 | 379.35 | 2.09 | 247.01 | 35.11 | 178.19 | 315.83 |
| 15-24 | 2020 | 18714 | 8485690 | 220.54 | 1.61 | 250.98 | 38.25 | 176.01 | 325.95 |
| 15-24 | 2021 | 2022 | 8290005 | 24.39 | 0.54 | 254.95 | 41.71 | 173.2 | 336.7 |
| 15-24 | 2022 | 10644 | 8245417 | 129.09 | 1.25 | 258.91 | 45.42 | 169.9 | 347.93 |
| 15-24 | 2023 | 33680 | 8131555 | 414.19 | 2.26 | 262.88 | 49.31 | 166.23 | 359.54 |
| 15-24 | 2024 | 22513 | 8071243 | 278.93 | 1.86 | 266.85 | 53.36 | 162.26 | 371.44 |
| 25-44 | 2005 | 6735 | 21804524 | 30.89 | 0.38 | 47.4 | 20.17 | 7.87 | 86.94 |
| 25-44 | 2006 | 6560 | 21840711 | 30.04 | 0.37 | 51.37 | 18.64 | 14.83 | 87.91 |
| 25-44 | 2007 | 9011 | 21841047 | 41.26 | 0.43 | 55.34 | 17.17 | 21.68 | 88.99 |
| 25-44 | 2008 | 14820 | 21919166 | 67.61 | 0.56 | 59.31 | 15.77 | 28.4 | 90.22 |
| 25-44 | 2009 | 10118 | 21654719 | 46.72 | 0.46 | 63.27 | 14.46 | 34.93 | 91.62 |
| 25-44 | 2010 | 21920 | 21469525 | 102.1 | 0.69 | 67.24 | 13.28 | 41.22 | 93.26 |
| 25-44 | 2011 | 13832 | 21345523 | 64.8 | 0.55 | 71.21 | 12.24 | 47.21 | 95.21 |
| 25-44 | 2012 | 11495 | 20561178 | 55.91 | 0.52 | 75.18 | 11.41 | 52.82 | 97.54 |
| 25-44 | 2013 | 32674 | 20294955 | 161 | 0.89 | 79.14 | 10.82 | 57.94 | 100.34 |
| 25-44 | 2014 | 9047 | 20113535 | 44.98 | 0.47 | 83.11 | 10.51 | 62.52 | 103.7 |
| 25-44 | 2015 | 36371 | 19960905 | 182.21 | 0.96 | 87.08 | 10.51 | 66.48 | 107.67 |
| 25-44 | 2016 | 15904 | 19798498 | 80.33 | 0.64 | 91.04 | 10.82 | 69.84 | 112.24 |
| 25-44 | 2017 | 15264 | 19662602 | 77.63 | 0.63 | 95.01 | 11.41 | 72.65 | 117.37 |
| 25-44 | 2018 | 18840 | 19549313 | 96.37 | 0.7 | 98.97 | 12.24 | 74.98 | 122.97 |
| 25-44 | 2019 | 26611 | 19408861 | 137.11 | 0.84 | 102.94 | 13.28 | 76.92 | 128.96 |
| 25-44 | 2020 | 15909 | 19320504 | 82.34 | 0.65 | 106.9 | 14.46 | 78.56 | 135.25 |
| 25-44 | 2021 | 1914 | 19220050 | 9.96 | 0.23 | 110.87 | 15.77 | 79.96 | 141.78 |
| 25-44 | 2022 | 12236 | 19383408 | 63.13 | 0.57 | 114.83 | 17.17 | 81.18 | 148.49 |
| 25-44 | 2023 | 34434 | 19246232 | 178.91 | 0.96 | 118.8 | 18.64 | 82.26 | 155.34 |
| 25-44 | 2024 | 28245 | 19022821 | 148.48 | 0.88 | 122.77 | 20.17 | 83.23 | 162.3 |
| 45+ | 2005 | 1627 | 18620767 | 8.74 | 0.22 | 15.38 | 7.8 | 0.1 | 30.65 |
| 45+ | 2006 | 1626 | 19320944 | 8.42 | 0.21 | 16.98 | 7.2 | 2.86 | 31.1 |
| 45+ | 2007 | 2158 | 18150823 | 11.89 | 0.26 | 18.58 | 6.63 | 5.58 | 31.58 |
| 45+ | 2008 | 3609 | 18938015 | 19.06 | 0.32 | 20.18 | 6.09 | 8.24 | 32.12 |
| 45+ | 2009 | 3271 | 19279537 | 16.97 | 0.3 | 21.78 | 5.59 | 10.83 | 32.73 |
| 45+ | 2010 | 7281 | 19896952 | 36.59 | 0.43 | 23.38 | 5.13 | 13.33 | 33.44 |
| 45+ | 2011 | 4939 | 20685540 | 23.88 | 0.34 | 24.98 | 4.73 | 15.71 | 34.25 |
| 45+ | 2012 | 4114 | 21276718 | 19.34 | 0.3 | 26.58 | 4.41 | 17.94 | 35.22 |
| 45+ | 2013 | 14287 | 22019347 | 64.88 | 0.54 | 28.18 | 4.18 | 19.99 | 36.38 |
| 45+ | 2014 | 4033 | 22691255 | 17.77 | 0.28 | 29.78 | 4.06 | 21.82 | 37.74 |
| 45+ | 2015 | 15896 | 23365606 | 68.03 | 0.54 | 31.38 | 4.06 | 23.42 | 39.34 |
| 45+ | 2016 | 7832 | 24032867 | 32.59 | 0.37 | 32.98 | 4.18 | 24.79 | 41.17 |
| 45+ | 2017 | 5225 | 24684674 | 21.17 | 0.29 | 34.58 | 4.41 | 25.94 | 43.22 |
| 45+ | 2018 | 8443 | 25297790 | 33.37 | 0.36 | 36.18 | 4.73 | 26.91 | 45.45 |
| 45+ | 2019 | 13299 | 25891671 | 51.36 | 0.45 | 37.78 | 5.13 | 27.73 | 47.83 |
| 45+ | 2020 | 8002 | 26456705 | 30.25 | 0.34 | 39.38 | 5.59 | 28.43 | 50.33 |
| 45+ | 2021 | 1324 | 26971514 | 4.91 | 0.13 | 40.98 | 6.09 | 29.03 | 52.92 |
| 45+ | 2022 | 6215 | 27825114 | 22.34 | 0.28 | 42.58 | 6.63 | 29.57 | 55.58 |
| 45+ | 2023 | 17620 | 28317269 | 62.22 | 0.47 | 44.17 | 7.2 | 30.06 | 58.29 |
| 45+ | 2024 | 16668 | 28831986 | 57.81 | 0.45 | 45.77 | 7.8 | 30.5 | 61.05 |
| All Ages | 2005 | 45827 | 63746522 | 71.89 | 0.34 | 109.15 | 27.8 | 54.66 | 163.63 |
| All Ages | 2006 | 46765 | 64296298 | 72.73 | 0.34 | 111.05 | 25.69 | 60.7 | 161.41 |
| All Ages | 2007 | 65544 | 62933515 | 104.15 | 0.41 | 112.96 | 23.66 | 66.58 | 159.34 |
| All Ages | 2008 | 89602 | 63721791 | 140.61 | 0.47 | 114.86 | 21.73 | 72.27 | 157.46 |
| All Ages | 2009 | 56626 | 63457439 | 89.23 | 0.37 | 116.77 | 19.93 | 77.7 | 155.84 |
| All Ages | 2010 | 116908 | 63646990 | 183.68 | 0.54 | 118.68 | 18.3 | 82.82 | 154.54 |
| All Ages | 2011 | 69798 | 64181000 | 108.75 | 0.41 | 120.58 | 16.87 | 87.51 | 153.65 |
| All Ages | 2012 | 77691 | 63319315 | 122.7 | 0.44 | 122.49 | 15.72 | 91.67 | 153.3 |
| All Ages | 2013 | 154430 | 63629640 | 242.7 | 0.62 | 124.39 | 14.9 | 95.18 | 153.61 |
| All Ages | 2014 | 41081 | 63930951 | 64.26 | 0.32 | 126.3 | 14.48 | 97.92 | 154.68 |
| All Ages | 2015 | 144951 | 64193418 | 225.8 | 0.59 | 128.2 | 14.48 | 99.83 | 156.58 |
| All Ages | 2016 | 63928 | 64405240 | 99.26 | 0.39 | 130.11 | 14.9 | 100.9 | 159.32 |
| All Ages | 2017 | 53961 | 64612113 | 83.52 | 0.36 | 132.01 | 15.72 | 101.2 | 162.83 |
| All Ages | 2018 | 86918 | 64798582 | 134.14 | 0.45 | 133.92 | 16.87 | 100.85 | 166.99 |
| All Ages | 2019 | 131157 | 64910184 | 202.06 | 0.56 | 135.83 | 18.3 | 99.97 | 171.68 |
| All Ages | 2020 | 72519 | 64984700 | 111.59 | 0.41 | 137.73 | 19.93 | 98.66 | 176.8 |
| All Ages | 2021 | 10617 | 64935348 | 16.35 | 0.16 | 139.64 | 21.73 | 97.04 | 182.23 |
| All Ages | 2022 | 46679 | 66060303 | 70.66 | 0.33 | 141.54 | 23.66 | 95.16 | 187.92 |
| All Ages | 2023 | 160839 | 65987498 | 243.74 | 0.61 | 143.45 | 25.69 | 93.09 | 193.8 |
| All Ages | 2024 | 103601 | 65912749 | 157.18 | 0.49 | 145.35 | 27.8 | 90.87 | 199.84 |
