# Supplementary material for: Epidemiological shift in age distribution of dengue in Thailand, 2005–2024
Source: PLoS Negl Trop Dis. 2026 Aug 31;20(8):e0014697. doi: 10.1371/journal.pntd.0014697 (PMC13552936; doi:10.1371/journal.pntd.0014697)
Supplement: Supplementary file 2 [file pntd.0014697.s002.docx]

**S2 Table. Adult (≥25-year) dengue case proportion by region and province, Thailand, 2005–2009 vs 2020–2024.**

Case-weighted percentage of dengue cases aged ≥25 years. Change is in percentage points (pp). *Z* is the Cochran-Armitage trend statistic across the four ordered periods (period scores 1–4); all provincial trends *P* < 0.001. Provinces are ordered by the size of the increase.

**Panel A. By region**

| Region | 2005–2009 (%) | 2020–2024 (%) | Change (pp) | CA *Z* |
| --- | --- | --- | --- | --- |
| North | 23.1 | 53.0 | 30.0 | 97.7 |
| Central | 19.1 | 36.3 | 17.3 | 80.1 |
| East | 23.8 | 40.7 | 16.9 | 53.3 |
| South | 19.3 | 35.9 | 16.6 | 60.5 |
| Northeast | 6.8 | 21.6 | 14.8 | 86.3 |
| Bangkok | 34.9 | 29.4 | -5.5 | 10.8 |

**Panel B. By province**

| Province | Region | 2005–2009 (%) | 2020–2024 (%) | Change (pp) | CA *Z* |
| --- | --- | --- | --- | --- | --- |
| Phayao | North | 21.2 | 54.7 | 33.6 | 23.66 |
| Phrae | North | 9.7 | 42.6 | 33.0 | 26.63 |
| Lampang | North | 20.0 | 48.8 | 28.8 | 23.73 |
| Nan | North | 30.2 | 58.3 | 28.1 | 15.56 |
| Surat Thani | South | 16.7 | 43.7 | 27.0 | 32.88 |
| Chiang Mai | North | 36.0 | 62.4 | 26.4 | 42.84 |
| Tak | North | 17.2 | 42.9 | 25.7 | 28.63 |
| Lamphun | North | 28.6 | 54.1 | 25.5 | 15.22 |
| P.Nakhon S.Ayutthaya | Central | 20.9 | 45.3 | 24.4 | 23.95 |
| Chiang Rai | North | 42.1 | 64.5 | 22.3 | 27.26 |
| Chanthaburi | East | 18.8 | 41.0 | 22.2 | 21.8 |
| Phitsanulok | North | 16.1 | 38.2 | 22.1 | 24.24 |
| Lop Buri | Central | 12.3 | 33.7 | 21.5 | 22.39 |
| Samut Songkhram | Central | 18.2 | 39.5 | 21.3 | 10.43 |
| Satun | South | 12.3 | 33.4 | 21.1 | 12.11 |
| Saraburi | Central | 13.1 | 34.2 | 21.1 | 18.66 |
| Prachin Buri | East | 15.1 | 36.2 | 21.1 | 21.23 |
| Mukdahan | Northeast | 10.0 | 30.6 | 20.6 | 15.31 |
| Phetchaburi | Central | 12.0 | 31.9 | 19.9 | 18.47 |
| Chachoengsao | East | 27.7 | 47.3 | 19.6 | 15.75 |
| Nakhon Si Thammarat | South | 13.6 | 33.1 | 19.5 | 31.15 |
| Rayong | East | 24.2 | 43.7 | 19.4 | 26.74 |
| Suphan Buri | Central | 17.7 | 36.7 | 19.0 | 18.68 |
| Nonthaburi | Central | 30.0 | 49.1 | 19.0 | 24.11 |
| Prachuap Khiri Khan | Central | 15.4 | 34.4 | 18.9 | 16.45 |
| Phatthalung | South | 18.7 | 37.5 | 18.8 | 19.06 |
| Nong Khai | Northeast | 6.0 | 24.6 | 18.6 | 17.15 |
| Roi Et | Northeast | 3.9 | 21.9 | 18.0 | 31.43 |
| Ang Thong | Central | 20.1 | 38.1 | 18.0 | 13.32 |
| Kamphaeng Phet | Central | 14.0 | 31.5 | 17.5 | 16.77 |
| Ranong | South | 19.7 | 37.3 | 17.5 | 7.46 |
| Uttaradit | North | 15.8 | 33.1 | 17.3 | 14.38 |
| Uthai Thani | Central | 15.2 | 32.5 | 17.3 | 11.87 |
| Pathum Thani | Central | 23.1 | 40.4 | 17.3 | 18.82 |
| Khon Kaen | Northeast | 5.6 | 22.7 | 17.1 | 25.8 |
| Phetchabun | North | 7.8 | 24.8 | 17.0 | 23.03 |
| Chai Nat | Central | 18.7 | 35.7 | 17.0 | 11.74 |
| Nakhon Ratchasima | Northeast | 8.6 | 25.6 | 17.0 | 31.2 |
| Phichit | Central | 17.9 | 34.8 | 16.9 | 14.88 |
| Phuket | South | 33.5 | 50.2 | 16.7 | 12.31 |
| Ratchaburi | Central | 15.6 | 32.1 | 16.6 | 25.27 |
| Sakon Nakhon | Northeast | 8.8 | 25.4 | 16.6 | 15.63 |
| Chumphon | South | 19.1 | 35.6 | 16.6 | 14.86 |
| Maha Sarakham | Northeast | 3.6 | 20.0 | 16.4 | 19.4 |
| Sukhothai | North | 11.4 | 27.7 | 16.3 | 15.1 |
| Yasothon | Northeast | 6.2 | 22.4 | 16.1 | 13.94 |
| Nakhon Nayok | Central | 21.1 | 37.2 | 16.1 | 9.56 |
| Kanchanaburi | Central | 11.6 | 27.5 | 15.9 | 16.88 |
| Loei | Northeast | 14.1 | 30.0 | 15.9 | 13.02 |
| Trang | South | 25.6 | 41.4 | 15.8 | 14.27 |
| Chon Buri | East | 28.9 | 44.3 | 15.4 | 25.87 |
| Krabi | South | 20.0 | 35.3 | 15.3 | 13.65 |
| Nakhon Sawan | Central | 18.0 | 33.3 | 15.3 | 22.2 |
| Nakhon Pathom | Central | 22.6 | 37.8 | 15.2 | 20.7 |
| Udon Thani | Northeast | 8.6 | 23.5 | 14.9 | 15.53 |
| Trat | East | 22.4 | 36.9 | 14.6 | 7.47 |
| Kalasin | Northeast | 5.5 | 19.7 | 14.2 | 19.24 |
| Songkhla | South | 16.2 | 29.7 | 13.4 | 20.88 |
| Amnat Charoen | Northeast | 6.9 | 20.1 | 13.3 | 11.33 |
| Narathiwat | South | 33.6 | 47.0 | 13.3 | 7.72 |
| Chaiyaphum | Northeast | 5.7 | 18.6 | 12.8 | 17.66 |
| Ubon Ratchathani | Northeast | 9.9 | 22.7 | 12.8 | 18.78 |
| Buri Ram | Northeast | 4.5 | 17.2 | 12.7 | 24.18 |
| Mae Hong Son | North | 42.0 | 54.7 | 12.7 | 9.57 |
| Sing Buri | Central | 19.3 | 32.1 | 12.7 | 3.97 |
| Si Sa Ket | Northeast | 7.9 | 19.0 | 11.1 | 20.76 |
| Samut Sakhon | Central | 25.0 | 35.6 | 10.6 | 12.06 |
| Surin | Northeast | 6.1 | 16.6 | 10.5 | 23.6 |
| Nong Bua Lam Phu | Northeast | 3.7 | 13.5 | 9.8 | 4.8 |
| Nakhon Phanom | Northeast | 7.8 | 17.4 | 9.6 | 3.74 |
| Sa Kaeo | East | 8.9 | 18.2 | 9.3 | 11.2 |
| Pattani | South | 14.5 | 22.5 | 7.9 | 9.33 |
| Phangnga | South | 33.6 | 41.3 | 7.7 | 3.77 |
| Samut Prakan | East | 28.8 | 35.9 | 7.1 | 8.53 |
| Yala | South | 24.7 | 30.1 | 5.4 | 3.42 |
| Bangkok | Bangkok | 34.9 | 29.4 | -5.5 | 10.77 |
